# Tailoring photonic metamaterial resonances for thermal radiation

**DOI:** 10.1186/1556-276X-6-549

**Published:** 2011-10-06

**Authors:** Peter Bermel, Michael Ghebrebrhan, Michael Harradon, Yi Xiang Yeng, Ivan Celanovic, John D Joannopoulos, Marin Soljacic

**Affiliations:** 1 Institute for Soldier Nanotechnologies, Massachusetts Institute of Technology, 77 Massachusetts Ave, Cambridge, MA 02139, USA

**Keywords:** metamaterials, photonic crystals, solar absorbers

## Abstract

Selective solar absorbers generally have limited effectiveness in unconcentrated sunlight, because of reradiation losses over a broad range of wavelengths and angles. However, metamaterials offer the potential to limit radiation exchange to a proscribed range of angles and wavelengths, which has the potential to dramatically boost performance. After globally optimizing one particular class of such designs, we find thermal transfer efficiencies of 78% at temperatures over 1,000°C, with overall system energy conversion efficiencies of 37%, exceeding the Shockley-Quiesser efficiency limit of 31% for photovoltaic conversion under unconcentrated sunlight. This represents a 250% increase in efficiency and 94% decrease in selective emitter area compared to a standard, angular-insensitive selective absorber.

**PACS: **42.70.Qs; 81.05.Xj; 78.67.Pt; 42.79.Ek

## 1 Background

Solar thermophotovoltaic (TPV) systems offer a distinct approach for converting sunlight into electricity [1-6]. Compared to standard photovoltaics, sunlight is not absorbed directly by a photovoltaic material, but is instead absorbed by a selective absorber. That selective absorber is thermally coupled to a selective emitter, which then thermally radiates electromagnetic radiation. The key challenge to making such a system efficient is achieving a relatively high temperature. Generally, this implies high optical concentrations [7]. However, one could consider whether there would be another way to concentrate heat in the selective absorber--without using optical concentrators at all. The key idea here is to replace the effect of optical concentration using a different method.

The most plausible approach to thermal concentration is angular selectivity--only allowing light to be absorbed within a small range of angles. The reason is that the apparent size of the sun is only a small fraction of the sky--approximately 1 part in 46,200 [8]. Several researchers have considered this in the context of photovoltaics [9] and thermophotovoltaics [6,10]. Metamaterials, such as photonic crystals, offer unprecedented control over wavelength- and angle-dependent absorptivity. In such systems, photon resonances can be tailored to target particular frequencies and conserved wavevectors to provide pinpoint control over thermal emission. Such an approach can be applied to create selective solar absorbing surfaces for applications such as solar thermal electricity, solar thermoelectrics, and solar thermophotovoltaics. The critical figure of merit is generally the fraction of incident solar radiation capable of being captured as heat. Typically, modest infrared emissivities put strict upper limits on the overall thermal transfer efficiency possible for the unconcentrated AM1.5 solar spectrum. However, carefully designed photonic metamaterials can strongly suppress thermal losses in the infrared.

**Figure 1:**
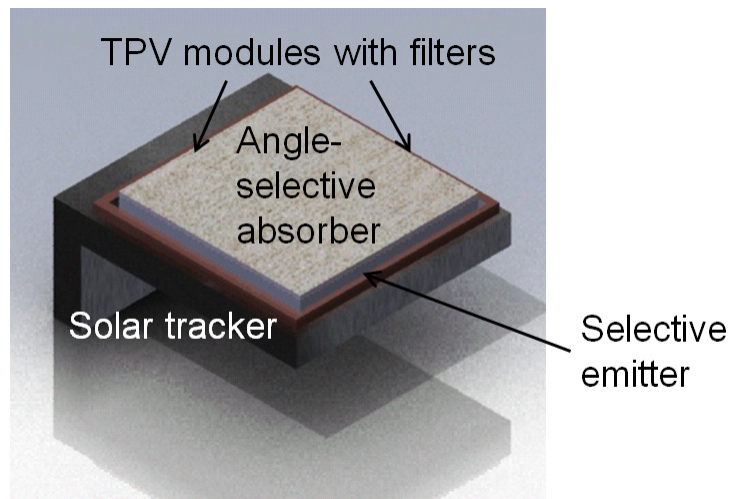
**Diagram of angle-selective solar thermophotovoltaic system**.

In this manuscript, we first characterize the performance of a standard solar TPV system without angular sensitivity, both in the ideal case and with a realistic amount of long-wavelength emissivity. We then quantify the improvement that can be achieved in a structure with long-wavelength emissivity using an optimized angle-sensitive design, as illustrated in Figure 1. We subsequently discuss design principles for structures with strong angular sensitivity, and present calculations on a structure more amenable to fabrication than previous 3D periodic designs [10], consisting of a 2D array of holes on the surface of tungsten.

The energy conversion efficiency of a solar TPV system such as in Figure 1 is defined to be [6]:

(1) $$\eta =\frac{I_{\text{m}}V_{\text{m}}}{CI_{\text{s}}A_{\text{s}}}$$

where *I*_m _and *V*_m _are the current and voltage of the thermophotovoltaic diode at the maximum power point, *C *is the concentration in suns relative to the solar constant *I*_s _(usually taken to be 1 kW/m^2^), and *A*_s _is the surface area of the selective solar absorber. This system can conceptually be decomposed into two halves: the selective solar absorber front end and the selective emitter plus TPV diode back end. Each half can be assigned its own efficiency: *η_t _*and *η_p_*, respectively.

The system efficiency can then be rewritten as:

(2) $$\eta =\eta_{t}\left(T\right)\eta_{p}\left(T\right)$$

where *T *is the equilibrium temperature of the selective absorber and emitter region. The efficiency of each subsystem can be further decomposed into its component parts. In particular, the selective solar absorber efficiency can be represented by [5,11]:

(3) $$\eta_{t}\left(T\right)=B\bar{\alpha }-\frac{\bar{\varepsilon }\sigma T^{4}}{CI_{\text{s}}}$$

where *B *is the window transmissivity, $\bar{\alpha }$ is the spectrally averaged absorptivity, $\bar{\varepsilon }$ is the spectrally averaged emissivity, and *σ *is the Stefan-Boltzmann constant.

The TPV diode back end efficiency can be represented by [6]:

(4) $$\eta_{p}=\frac{I_{\text{m}}V_{\text{m}}}{{\bar{\varepsilon }}_{\text{E}}A_{\text{E}}\sigma T^{4}}$$

where ${\bar{\varepsilon }}_{\text{E}}$ and *A*_E _are the effective emissivity and area of the selective emitter, respectively.

## 2 Results and discussion

**Figure 2:**
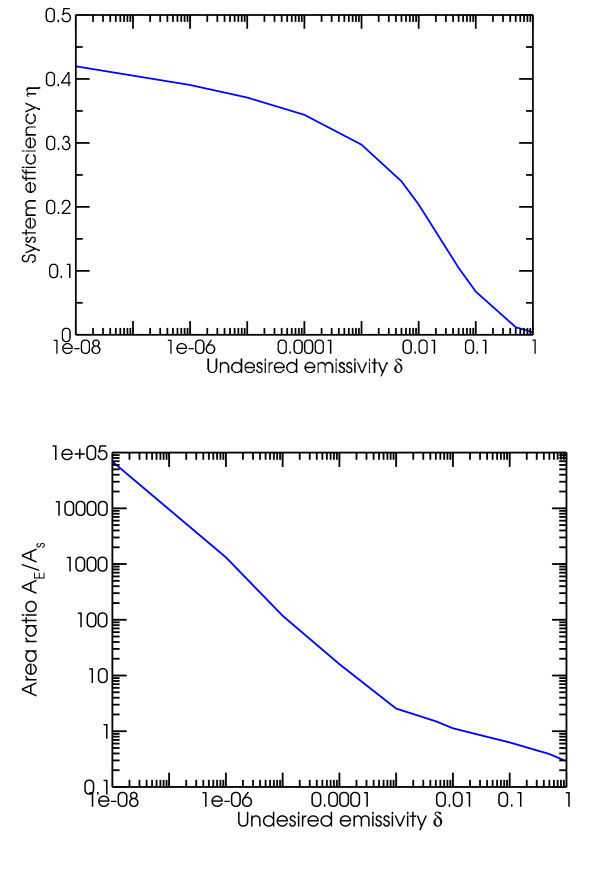
**For an ideal solar TPV system with unwanted emissivity *δ***: **a **system efficiency versus *δ *and **b **area ratio for selective emitter to selective absorber versus *δ*.

We can begin by considering the situation where absorptivity for both the selective absorber and emitter is unity within a certain frequency range, and *δ *otherwise. The ranges for the selective absorbers and emitters are optimized separately, and the lower end of the selective emitter frequency range equals the TPV diode bandgap frequency *ω_g_*. If we consider the case of unconcentrated sunlight, the limit *δ *→ 0 implies a decoupling between the selective absorber and emitter, where the selective absorber is kept relatively cool to maximize *η_t_*, while the selective emitter acts as if it were much hotter with a bandgap frequency *ω_g _*well over the blackbody peak predicted by Wien's law. However, this also leads to declining effective emissivity ${\bar{\varepsilon }}_{\text{E}}\propto \delta$, and thus *A*_E_/*A*_s _∝ 1/*δ*. This expectation is supported by the numerical calculations in Figure 2 (see the Methods sections for details), which demonstrate both that efficiency slowly increases with decreasing *δ*, while the area ratio increases rapidly as 1/*δ*. Clearly the limit where *δ *→ 0 and *A_E_*/*A_s _*→ ∞ is unphysical, both because the time to establish equilibrium in an arbitrarily large system is arbitrarily long, and a perfectly sharp emissivity cutoff requires a step function in the imaginary part of the dielectric constant of the underlying material. However, the latter is inconsistent with the Kramers-Kronig relations for material dispersion, which derive from causality [12].

**Figure 3:**
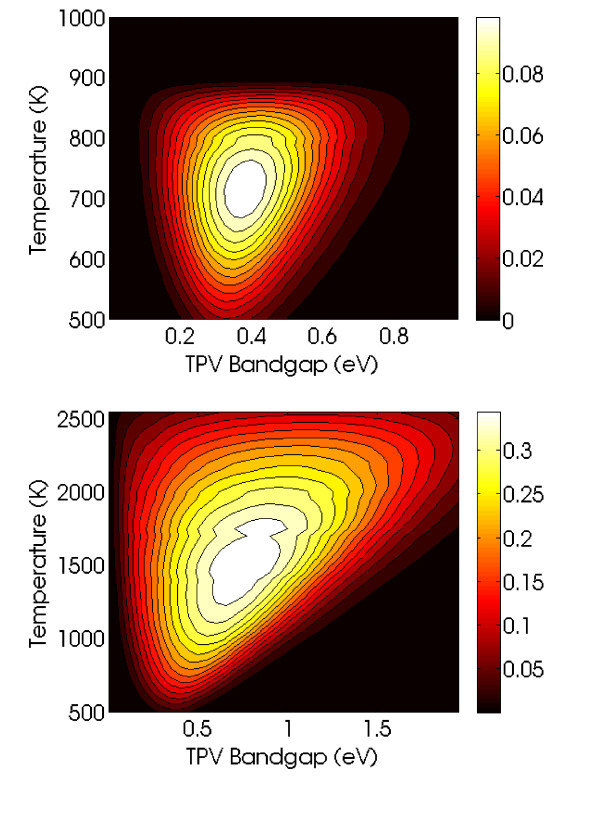
**Solar TPV system efficiency**: **a **without angular selectivity, **b **with optimized angular selectivity of functional form given in Equation 5.

Based on a comprehensive review of selective solar absorbers [13], typical spectrally averaged selective solar absorber emissivities $\bar{\varepsilon }$ are about 0.05 at temperatures of approximately 373 K. Assuming *δ *= 0.05 as well, this implies a maximum system efficiency of 10.5% (*T *= 720 K, *η_t _*= 0.6937, *η_p _*= 0.1510, *A_E_/A_s _*= 0.75), as illustrated in Figure 3a. While a physically relevant result, this efficiency is unfortunately less than a quarter of the asymptotic efficiency calculated above as *δ *→ 0.

**Figure 4:**
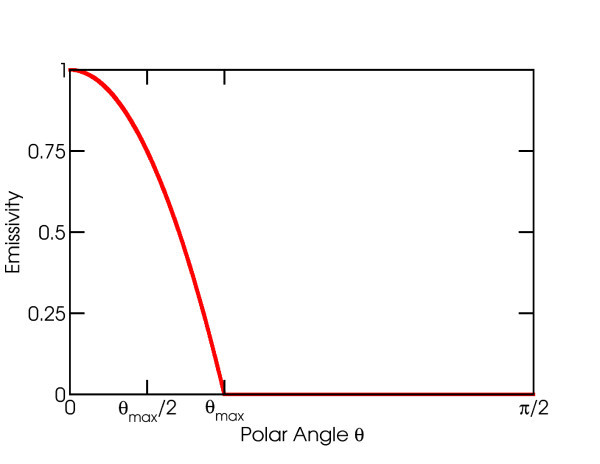
**Schematic diagram of the emissivity as a function of angle for all wavelengths**.

To bridge the gap between performance of solar TPV in the cases where *δ *= 0.05 and *δ *→ 0, we can employ a combination of wavelength and angle selectivity. It has been shown in a large number of previous publications that absorption can be made to peak at a certain target angle or wavevector, over a certain range of wavelengths. While an exact analytical expression is often lacking, it generally resembles a top hat function in wavelength space, and a local maximum in the angular dimension [14,15]. Since local maxima can be approximated as inverted parabolas, the analytical expression we use is as follows [14,15]:

(5) $$\varepsilon \left(\omega ,\;\theta \right)=\left[1-{\left(\theta ∕\theta_{\max}\right)}^{2}\right]\left[\delta +\left(1-\delta \right)\Pi_{\omega_{1},\omega_{2}}\left(\omega \right)\right],$$

where $\Pi_{\omega_{1},\omega_{2}}\left(\omega \right)$ is the top hat function, equal to 1 if *ω*_1 _<*ω < ω*_2 _and 0 otherwise. This definition is illustrated in Figure 4 for frequencies within the window of the top hat.

**Figure 5:**
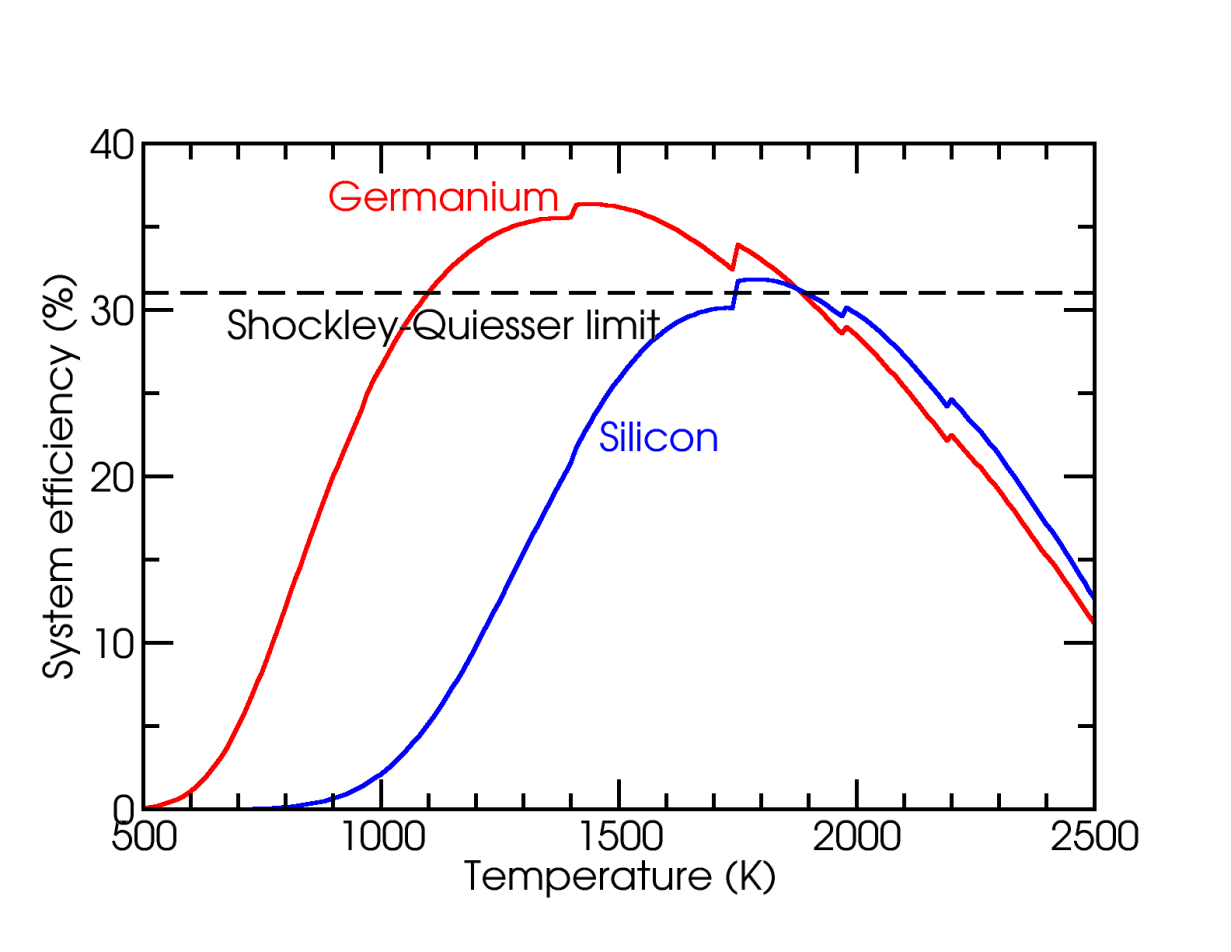
**Solar TPV system efficiency as a function of operating temperature for germanium and silicon with unconcentrated sunlight**. Both can exceed the Shockley-Quiesser limit at certain operating temperatures.

The system efficiency of our angle-selective design was determined by inserting Equation 5 into Equation 3, then multiplying with the TPV diode back end efficiency of Equation 4. Optimizing over the following parameters--cutoff frequencies, acceptance angles, TPV bandgap and temperature--yields the results in Figure 3b, where the maximum efficiency is 37.0% (*T *= 1, 600 K, *η_t _*= 0.7872, *η_p _*= 0.4697, *A*_E_/*A*_s _= 0.05). This is 3.5 times higher than our previous result, and fairly close to the asymptotic limit where *δ *→ 0 from before, without the physically unreasonable requirement of a perfectly sharp emissivity cutoff (which is inconsistent with causality). This result also exceeds the Shockley-Quiesser limit for photovoltaic energy conversion in unconcentrated sunlight of 31% efficiency [8]. Furthermore, as illustrated in Figure 5, photovoltaic diodes made from group IV compounds such as silicon and germanium have bandgaps that would allow for the system to continue to exceed the Shockley-Quiesser limit. Finally, the much lower area ratio *A*_E_/*A*_s _= 0.05 implies that the angle-selective solar absorber illustrated in Figure 1 would serve as a sort of thermal concentrator, thus allowing for much less thermophotovoltaic area to be used compared to previous designs in the literature.

**Figure 6:**
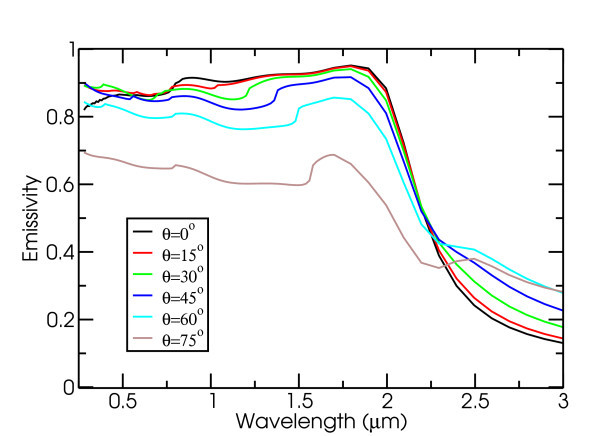
**Emissivity spectra for 2D periodic arrays of cylindrical holes in single crystal tungsten at various angles (*a *= 800 nm, *r *= 380 nm, and *d *= 3.04 *μ*m**. Notice that the average emissivity gradually decreases with increasing angle away from normal incidence.

Finally, we consider reasonable metamaterial designs for achieving the desired effective emissivity in Equation 5. Most structures with nanoscale features on the surface in both directions have potential to exhibit strong angular sensitivity. The specific structure we examined is a 2D array of cylindrical holes in single-crystal tungsten, as discussed in [5]. In Figure 6, using numerical techniques described in the Methods section, we show that an optimal structure with period 800 nm, hole radius 380 nm, and hole depth 3.04 *µ*m exhibits decreasing average emissivity with increasing angle away from normal incidence. In particular, at a 75° angle, the average emissivity for wavelengths from 400 nm to 2 *μ*m is 30% lower than at normal incidence. Overall, for an absorber in unconcentrated sunlight held at 400 K, the spectrally averaged absorptivity $\bar{\alpha }=0.867$, while the spectrally average emissivity $\bar{\varepsilon }=0.073$. This results in a projected thermal transfer efficiency *η_t _*= 0.750. Such a result compares favorably with previously proposed selective absorber designs, such as a germanium with a silver back and an anti-reflection coating, with a projected thermal transfer efficiency of 0.678 under identical conditions [5]. Additionally, increasing the operating temperature to 1,000 K and employing 100 sun concentration (e.g., with a parabolic trough) yields a projected thermal transfer of 0.741; again, above a semiconductor-based design with an anti-reflection coating, displaying a thermal transfer efficiency of 0.710 under identical conditions [5]. Clearly, suppressing off-angle emission with relatively simple structures such as 2D arrays of holes in tungsten can give rise to improved spectrally selective performance. Future work should focus on modifying these structures to narrow the acceptance angles. This approach should allow one to achieve record-setting thermal transfer efficiencies for selective solar absorbers.

## 3 Conclusions

It was found that although in principle solar thermophotovoltaic systems in unconcentrated sunlight can exceed efficiencies of 42%, achieving such performance requires suppression of emissivities to unreasonably low levels. Conventional materials with undesired emissivities of 0.05 display much lower efficiencies of 10.5%. However, most of the theoretically allowed performance can be restored by introducing angular selectivity of the assumed form in Equation 5, with up to 37% overall system efficiency. The system also acts as a thermal concentrator, with receiver areas 20 times larger than the emitter areas. Finally, we considered 2D arrays of nanoscale cylindrical holes in single crystal tungsten as a candidate metamaterial for angle-selective operation, and found the optimal design parameters to be a period of 800 nm, a radius of 380 nm, and a depth of 3.04 *μ*m, with a thermal transfer efficiency of 75.0% in unconcentrated sunlight at 400 K.

## 4 Methods

Simulations of electromagnetic properties were conducted following the same methods as outlined in [5]. We employ a finite difference time-domain (FDTD) simulation [16] implemented via a freely available software package developed at MIT, known as MEEP [17]. A plane wave is sent from the normal direction and propagated through space. On each grid point of a flux plane defined at the front and back of the computational cell, the electric and magnetic fields are Fourier-transformed via integration with respect to preset frequencies at each time-step. At the end of the simulation, the Poynting vector is calculated for each frequency and integrated across each plane, which yields the total transmitted and reflected power at each frequency [17]. The dispersion of tungsten is captured via a Lorentz-Drude model [18]. Apart from the approximations of material dispersions and grid discretization, these calculation methods are exact.

The emissivity of each structure can be calculated from the absorptivity computed above via Kirchhoff's law of thermal radiation, which states that the two quantities must be equal at every wavelength for a body in thermal equilibrium [19].

The system efficiency is calculated from numerical integration (via the trapezoidal rule) of Eqs. 3 and 4, and taking their product as in Equation 2. It can then be globally optimized through the application of the multi-level single-linkage (MLSL), derivative-based algorithm using a low-discrepancy sequence (LDS) [20]. This algorithm executes a quasi-random (LDS) sequence of local searches using constrained optimization by linear approximation (COBYLA) [21], with a clustering heuristic to avoid multiple local searches for the same local minimum. We verified that other global search algorithms, such as DIRECT-L [22], yield similar results. This ability to directly utilize and compare multiple optimization packages on the same problem is provided by the NLopt package, written by Prof. Steven G. Johnson and freely available at http://ab-initio.mit.edu/nlopt.

## Competing interests

The authors declare that they have no competing interests.

## Authors' contributions

PB calculated the figure of merit and drafted the manuscript. MG developed optimization code utilizing NLopt. MH performed transfer matrix simulations of the 2D PhC structures; YY confirmed the observed behaviors in MEEP. MS developed the concept of 2D angular-selective absorbers; IC suggested focusing particularly on tungsten 2D photonic crystals. JDJ determined the appropriate simulation methods for this study. All authors read and approved the final manuscript.

## References

[B1] Spirkl W Ries H Solar thermophotovoltaics: an assessment J Appl Phys 1985 57 4409 10.1063/1.334602

[B2] Luque A Solar Thermophotovoltaics: Combining Solar Thermal and Photovoltaics AIP Conf Proc 2007 890 3

[B3] Datas A Algora C Corregidor V Martin D Bett A Dimroth F Fernandez J Optimization of Germanium Cell Arrays in Tungsten Emitter-based Solar TPV Systems AIP Conf Proc 2007 890 227

[B4] Rephaeli E Fan S Absorber and emitter for solar thermophotovoltaic systems to achieve efficiency exceeding the Shockley-Queisser limit Opt Express 2009 17 15145 10.1364/OE.17.015145 19687992

[B5] Bermel P Ghebrebrhan M Chan W Yeng YX Araghchini M Hamam R Marton CH Jensen KF Soljacic M Joannopoulos JD Johnson SG Celanovic I Design and global optimization of high-efficiency thermophotovoltaic systems Opt Express 2010 18 A314 10.1364/OE.18.00A314 21165063

[B6] Datas A Algora C Detailed balance analysis of solar thermophotovoltaic systems made up of single junction photovoltaic cells and broadband thermal emitters Sol Energy Mater Sol Cells 2007 94 2137

[B7] Harder N Wurfel P Theoretical limits of thermophotovoltaic solar energy conversion Semicond Sci Technol 2003 18 S151 10.1088/0268-1242/18/5/303

[B8] Henry C Limiting efficiencies of ideal single and multiple energy gap terrestrial solar cells J Appl Phys 1980 51 4494 10.1063/1.328272

[B9] Goetzberger A Goldschmidt J Peters M Loper P Light trapping, a new approach to spectrum splitting Sol Energy Mater Sol Cells 2008 92 1570 10.1016/j.solmat.2008.07.007

[B10] Florescu M Lee H Puscasu I Pralle M Florescu L Ting DZ Dowling JP Improving solar cell efficiency using photonic band-gap materials Sol Energy Mater Sol Cells 2007 91 1599 10.1016/j.solmat.2007.05.001

[B11] Zhang QC High efficiency Al-N cermet solar coatings with double cermet layer film structures J Phys D Appl Phys 1999 32 1938 10.1088/0022-3727/32/15/324

[B12] Ashcroft NW Mermin ND Solid State Physics 1976 Philadelphia: Holt Saunders

[B13] Kennedy C Review of mid- to high-temperature solar selective absorber materials Tech. Rep. TP-520-31267, National Renewable Energy Laboratory 2002

[B14] Menzel C Helgert C Upping J Rockstuhl C Kley EB Wehrspohn R Pertsch T Lederer F Angular resolved effective optical properties of a Swiss cross metamaterial Appl Phys Lett 2009 95 131104 10.1063/1.3238554

[B15] Chutinan A John S Light trapping and absorption optimization in certain thin-film photonic crystal architectures Phys Rev A 2008 78 023825

[B16] Taflove A Hagness SC Computational electrodynamics 2000 2 Norwood: Artech House

[B17] Oskooi AF Roundy D Ibanescu M Bermel P Joannopoulos JD Johnson SG MEEP: A flexible free-software package for electromagnetic simulations by the FDTD method Comput Phys Commun 2010 181 687 10.1016/j.cpc.2009.11.008

[B18] Rakic A Djurisic A Elazar J Majewski M Optical properties of metallic films for vertical-cavity optoelectronic devices Appl Opt 1998 37 5271 10.1364/AO.37.005271 18286006

[B19] Rybicki G Lightman A Radiative processes in astrophysics 1979 New York: Wiley

[B20] Kucherenko S Sytsko Y Application of deterministic low-discrepancy sequences in global optimization Comput Optim Appl 2005 30 297 10.1007/s10589-005-4615-1

[B21] Powell M Advances in optimization and numerical analysis 1994 Dordrecht: Kluwer Academic 51 67

[B22] Gablonsky JM Kelley CT A locally-biased form of the DIRECT algorithm J Global Optim 2001 21 1 27 10.1023/A:1017930332101

